# Aging Mouse Models Reveal Complex Tumor-Microenvironment Interactions in Cancer Progression

**DOI:** 10.3389/fcell.2018.00035

**Published:** 2018-03-29

**Authors:** Hidetoshi Mori, Robert D. Cardiff, Alexander D. Borowsky

**Affiliations:** ^1^Center for Comparative Medicine, University of California, Davis, Davis, CA, United States; ^2^Department of Pathology and Laboratory Medicine, School of Medicine, University of California, Davis, Davis, CA, United States

**Keywords:** genetically engineered mouse models (GEMMs), gene knockout mice, aging, mammary tumorigenesis, mouse strain

## Abstract

Mouse models and genetically engineered mouse models (GEMM) are essential experimental tools for the understanding molecular mechanisms within complex biological systems. GEMM are especially useful for inferencing phenocopy information to genetic human diseases such as breast cancer. Human breast cancer modeling in mice most commonly employs mammary epithelial-specific promoters to investigate gene function(s) and, in particular, putative oncogenes. Models are specifically useful in the mammary epithelial cell in the context of the complete mammary gland environment. Gene targeted knockout mice including conditional targeting to specific mammary cells can reveal developmental defects in mammary organogenesis and demonstrate the importance of putative tumor suppressor genes. Some of these models demonstrate a non-traditional type of tumor suppression which involves interplay between the tumor susceptible cell and its host/environment. These GEMM help to reveal the processes of cancer progression beyond those intrinsic to cancer cells. Furthermore, the, analysis of mouse models requires appropriate consideration of mouse strain, background, and environmental factors. In this review, we compare aging-related factors in mouse models for breast cancer. We introduce databases of GEMM attributes and colony functional variations.

## Preamble

Since the introduction of gland cleared mammary fat pad by DeOme and associates (Deome et al., 1959), mammary gland and mammary cell transplantation has been used to study aging in the mammary gland. One of the initial observations was that normal mammary epithelium had a limited capacity to fill the mammary fat pad and the original mammary epithelium would not grow after five or so serial transplants (Daniel et al., 1968). Serial transplant senescence defines a property of normal mammary epithelium, with “immortalization” or non-senescence proof of a neoplastic transformation (Daniel et al., 1968, 1975). Subsequently, this “test-by-transplantation” became the basis for the early descriptions of mammary gland stem cells their “committed” intermediates (Smith and Medina, 1988, 2008). This experiment laid the foundation for modern stem cell research. Although endocrine influence on the mammary gland was well-understood at the time, additional environmental factors were somewhat ignored (Young et al., 1971; Daniel et al., 1983) and not widely applied to problems in neoplasia.

Genetic engineering technology brought a new scientific generation into the study of experimental mammary tumorigenesis. The literature produced deals only indirectly with the problems of aging and mouse mammary tumorigenesis. Our objective is to organize and discuss this abundant data set specifically in the context of aging-related cancer.

## Background

Breast cancer is an aging-related disease. According to American Cancer Society (www.cancer.org), multiple factors have been identified as risk factors. These factors include *aging*, gene mutations, ethnicity, breast tissue density, early menarche, menopause after age 55, exposure to certain chemicals and life style related factors (e.g., parity, breast feeding, birth control, hormone therapy, drinking alcohol, and obese). Each of the factors indicated above are also linking to aging. For example, first birth at an older age results in a higher risk (MacMahon et al., 1970) and the risk after parity is sustained in a woman who has her first pregnancy at an older age (Albrektsen et al., 2005; Meier-Abt and Bentires-Alj, 2014). Like the famous “nun study” suggesting that parity was protective, more recent studies comparing nulliparous and parous Norwegian women demonstrated that parity has a slight protective effect that increases with age (Albrektsen et al., 2005). The mechanism of increased cancer risk related to these proven epidemiological factors has been extensively investigated and debated. Clearly, endocrine changes with time affects incidence, but also raises a central conundrum of breast cancer risk—why does cancer risk increase with advancing age *even as endogenous estrogen levels drop after menopause?* And, for our purposes in this review, *do mice model this change?* Mice do not go through a “menopause” *per se*, but their reproductive capacity and hormonal levels, including estrogen, decline around 1 year of age (Nelson et al., 1982; Finch et al., 1984). Perhaps the better question is—*do mice live long enough to get mammary cancer?* This is where genetic engineering can help. Originally construed as a simple, cell intrinsic, proof of oncogene function, like a modern Koch's postulate for oncogenes, these genetic alterations, in many cases, instead result in a mild acceleration of the risk for developing mammary tumors. These genotypes are the important GEMM for studying breast cancer risk, risk modifiers, and aging.

A wide variety of GEMM have been generated to study beast and other cancers. Strong mammary epithelial specific-promoters driving potent oncogenes are most commonly used, with the obvious practical benefit of rapid and highly penetrant cancer development—a clear practical benefit to experimental design and execution (Borowsky, 2011). Mouse mammary tumor virus long terminal repeat (*MMTV-LTR*) (note: this construct does not include other viral elements, such as T antigens, and is not transforming by itself) and milk protein promoters such as whey acidic protein (*Wap*) and beta lactoglobulin (*Blg*) promoters are the most extensively used for transducing breast cancer-related genes in mouse mammary epithelial cells. However, the target molecule under *MMTV-LTR* promotion is transduced even in early developmental stages (Wagner et al., 2001), and most tumors with the *MMTV-LTR* as a promoter induce strong growth signals at a relatively early age in the mouse life span. On the other hand, *Wap* RNA expression is up-regulated by lactogenic hormones and is induced during the late stages of parturition and lactation (Pittius et al., 1988). This trait makes the *Wap* promoter a useful tool for analyzing the pregnancy-dependent effects. However, the transgene under *Wap* promoter activity is also activated in nulliparous mice and in early pre-lactation (Pittius et al., 1988) as well as during the early stages of involution (Wagner et al., 1997). The promoter activity is, however, the highest during pre-lactation/lactation.

Tumors arising in GEMM with transgenes constructed from strong epithelial promoters can be difficult to interpret. The promoters themselves are hormonally regulated, so that studying hormone blockers or depletion in these models can yield the trivial mechanistic result of down-regulation of the transgene. There is also the problem of simultaneous development and progression of multiple primary tumors, sometimes making the progression of a specific, single tumor impossible to study.

Gene knockout (GKO) mice also have been generated for breast cancer research and exhibit differences in mammary gland development and/or tumorigenesis. The GKO mice are generally designed to test for tumor suppressor type of genes. Therefore, the GKO does not usually result in the neoplastic alteration by itself. However, tumor suppressor GKOs often result in dramatic changes in tumor kinetics when crossed with an oncogenic transgene GEMM. Conditional gene knockouts using Cre-LOX technology and using mammary luminal specific (*MMTV-LTR-Cre*; *Wap-Cre*; *Blg-Cre*) or basal specific (*K14*- or *K5-Cre*) targeting are useful to focus on the molecular involvement in epithelial cells but may not reflect the importance of changes in the microenvironment during breast cancer progression.

The analysis of aging effects in breast cancer progression requires an understanding of senescence of any cell in the microenvironment as well as the cancer cell. Therefore, such phenomena as the immune cell activities on clearing damaged senescent cells must be considered. However, very little is known about how these microenvironment and immune cell activities are involved in GEMM. Variations in phenotypes depending on the mouse strain's background which also limits comparisons with other models. Further, the “environmental” factors, such as mouse housing, cycles of circadian rhythm, diet, viral—and bacterial-infections, can also affect the phenotype. However, environmental variables are almost completely ignored when models are compared. These environmental factors are also known as modifiers for immune cell activities and are important for immune cell-related aging. In this report, we introduce GEMM for breast cancer research which might be useful tools for analyzing the effects of aging and microenvironment.

## Aging parameters in GEMM

Aging in the mouse has been extensively described and discussed in literature and in monographic compendiums about pathobiology (Carlton et al., 2001) and physiology (Flurkey et al., 2007). The pathobiology has been updated for application to GEMM (Brayton et al., 2012). Numerous biomarkers for mouse aging and senescence have been described and applied. For example, the use of T-cell populations and IGF-1 levels have been widely used but other markers are also quite useful (Flurkey et al., 2007).

Endocrine factors have also been analyzed for aging related study in mouse (Flurkey et al., 2007). The status of ovarian hormone production is key to understanding aging-related hormone receptor positive neoplasms in mouse and human mammary neoplasms. Although estrogen receptor (ER) positive mammary tumors in GEMM have been analyzed in prior studies (reviewed in Dabydeen and Furth, 2014), not all models have been tested for hormone dependent- and independent-tumors actions using ovariectomy or hormone disrupting chemicals. Ovarian ablation experiments are important in GEMM because aged mice are not anovulatory as in humans (see the section below: “Aging in the human breast and mouse mammary gland and microenvironment”).

In the absence of biomarker data, life span is used for comparing potential aging effects on tumorigenesis in GEMM. Since the standard measurement of biomarkers have not been applied to GEMM of breast cancer and few, if any, laboratories keep colonies with sufficient populations to have accurate length of life data. We have arbitrarily adapted Harrison's life stages graph to estimate Mature (3–6 months), Middle Age (10–14 months), and Old (18–24 months) (Flurkey et al., 2007). Further, we have converted the numbers from months to weeks or days. The only data available, in most tumor related papers, is the T50 of tumorigenesis. These data can be misleading because of the chimeric populations used in some studies. However, the T50 is a useful metric for comparison.

## Mammary tumors in *MMTV-LTR* and *Wap* promoter driven mouse models

**Figure 1:**
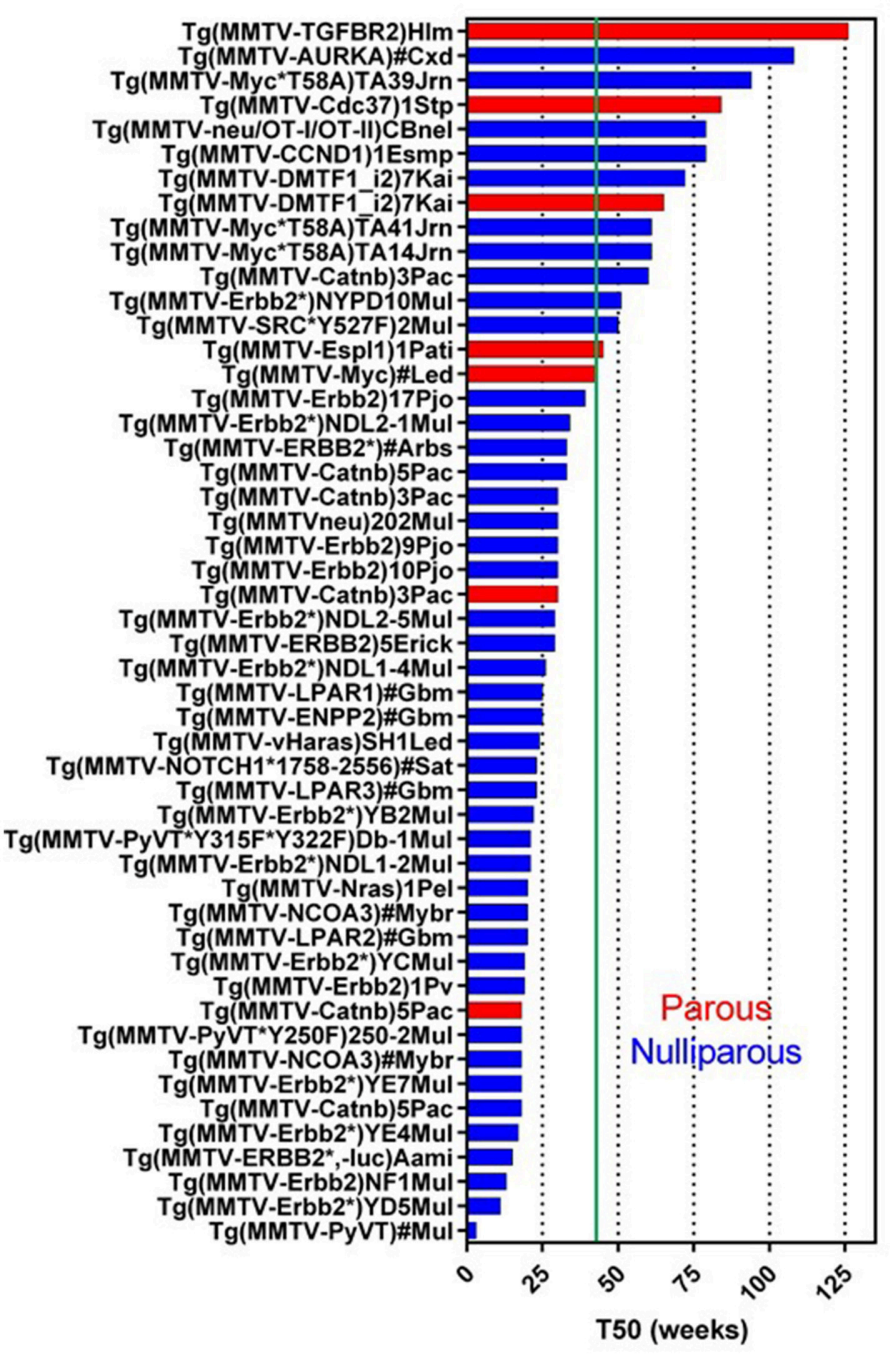
Tumor latency in *MMTV-LTR* driven GEMM. A graph indicates a tumor latency (T50, weeks) in each *MMTV-LTR* driven GEMM. GEMM in Mouse Gemone Informatics (http://www.informatics.jax.org/) are shown. Blue and red bars indicate nulliparous and multiparous, respectively. Green line indicates 43 weeks (10 months) when the mouse is in age equivalent in mid-age human (Flurkey et al., 2007). If each publication described T50 or a graph for percentage of tumor free, T50 is indicated as weeks. If the publication does not indicate these parameters but has enough information to predict T50, indicated T50 were calculated. *MMTV-LTR* driven GEMM, which does not have sufficient information to calculate T50 or have other information (e.g., non-palpable tumor but microscopic observation for hyperplastic region), are summarized in additional Supplementary Data Sheet 1.

*MMTV-LTR* was first used to generate Tg(*MMTV-Myc*) in 1984 (Stewart et al., 1984). The activity of this promoter is sensitive to progesterone and dihydrotestosterone but not to estrogen (Otten et al., 1988). It can also be affected by the combinations of other factors, such as cell density, nutrition, growth factor, and hormones (Young et al., 1975; Cardiff et al., 1976). Currently, 114 GEMM lines targeting 45 genes (except for *tTA, rtTA*, and *luc*) have been registered in Mouse Genome Informatics (MGI) (Figure 1) (Bult et al., 2015; Blake et al., 2017; Finger et al., 2017). Of these 45 genes, human or mouse derived, 27 genes promoted by the *MMTV-LTR* developed mammary tumors. Whereas the median tumor latency time (a.k.a. median tumor-free latency; T50) is not always indicated or reported and the estimated T50 is variable ranging from 4 weeks to more than a year (52 weeks). Since *MMTV-LTR* is also active in embryonic tissues, the gene induction can be higher in developmental stages of mammary gland growth (Wagner et al., 2001). Transgenes with strong signaling inducing activity (i.e., PyMT, Erbb2, Catnb, Nras, Haras) form palpable tumors at relatively young ages. However, interestingly, the T50 in several *MMTV-LTR* driven GEMM are longer than a year. Remember that according to Flurkey et al. mice aged 10–14 months are equivalent to 38–47 years middle-aged human, and 18–24months are equivalent to 56–69 years older human (Flurkey et al., 2007).

In *MMTV-LTR* driven tumor-forming GEMM (Figure 1), mice with T50 longer than 10 months (43 weeks) or older number 13 including: *Myc* (Cardiff et al., 1991), *Espl1* (Mukherjee et al., 2014), constitutively active *SRC* (Y527F) (Webster et al., 1995), auto-phosphorylation mutant *Erbb2* (NYPD) (Dankort et al., 2001), *Catnb* (Imbert et al., 2001), *DMP1*β (Maglic et al., 2015), *CCND1* (Wang et al., 1994), activated rat *neu* oncogene tagged with CD4 and CD8 epitopes (OT-I/OT-II) (Wall et al., 2007), *Cdc37* (Stepanova et al., 2000), *Myc* mutant insensitive to KRas signal (T58A) (Andrechek et al., 2009), *AURKA* (Wang et al., 2006) and dominant negative type II TGFb receptor (*TGFBR2*) (Gorska et al., 1998).

Some models, when analyzed, demonstrate that parity facilitates tumorigenesis. *MMTV-Catnb* (Imbert et al., 2001), *MMTV-Myc* (Stewart et al., 1984; Cardiff et al., 1991), and *MMTV-DMTF1* (Maglic et al., 2015) in parous mice have shorter T50 than nulliparous females. The *MMTV-Espl* GEMM forms tumor only with pregnancy (Mukherjee et al., 2014). Similarly, *MMTV-PTGS2* nulliparous female mice do not develop tumors in, but 85% of multiparous female mice develop tumors (Liu et al., 2001).

Even GEMMs without tumor, crossbred to a GEMM with mammary tumors often results in different tumor kinetics. For example, the following crosses all resulted in accelerated tumor development: *MMTV-MAT* × *MMTV-neu* (Rudolph-Owen et al., 1998)*, MMTV-Vegfa* × *MMTV-neuYD* (Oshima et al., 2004), MMTV-Akt1^*^T308D^*^S473D × *MMTV-PyMT* (Hutchinson et al., 2001). In contrast, *MMTV-DTX1* × *MMTV-HRas* (or *MMTV-cMyc*) bigenic mice had better survival (Kiaris et al., 2004). Some GEMM publications which do not have sufficient information to estimate T50, are indicated in Supplementary Data Sheet 1.

**Figure 2:**
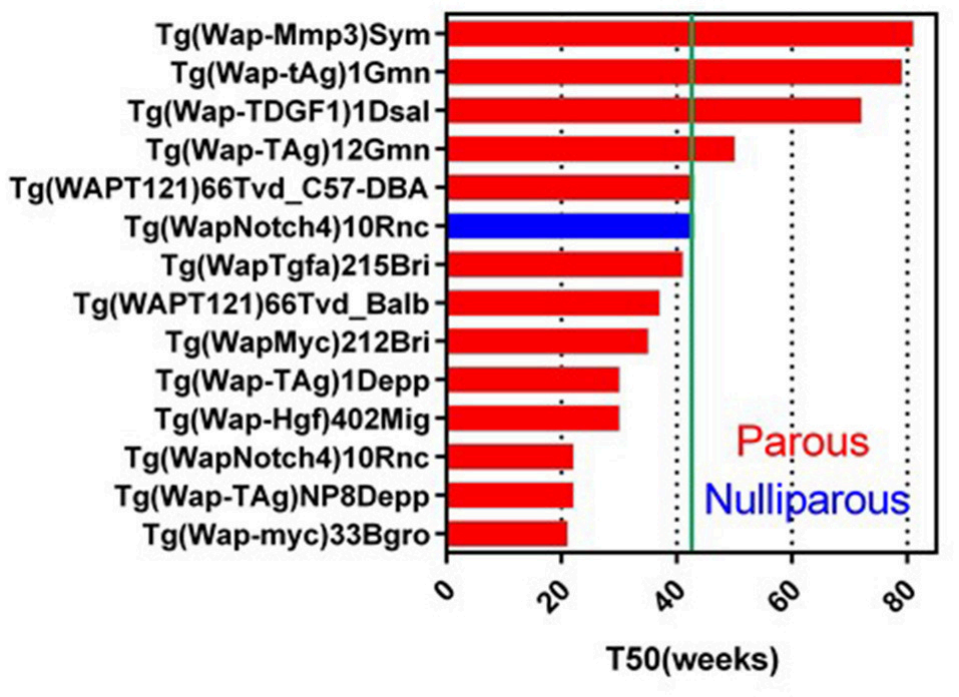
Tumor latency in *Wap*-promoter driven GEMM. A graph indicates a tumor latency (T50, weeks) in each *Wap*-promoter driven GEMM. Blue and red bars indicate nulliparous and multiparous, respectively. Green line indicates 43 weeks (10 months). As same as *MMTV-LTR* driven GEMM, if each publication does not indicate T50, the T50 is estimated from the information. Additional information for GEMM not listed on this graph is available in Supplementary Data Sheet 1.

*Wap*-promoter driven mouse model was first used to make *Wap-HRAS* (Andres et al., 1987; Nielsen et al., 1991). In 2017, 25 GEMM lines targeting 16 genes are registered in MGI. *Wap* promoter activity has been used to transactivate target cDNA in mammary epithelial cells during pre-lactation and lactation. These models might be useful for the study of parity-dependent tumorigenesis. In these GEMM, 13 genes transduced with *Wap*-promoter formed mammary tumor (Figure 2). Although the pregnancy usually occurs in younger, reproductive age mice, the T50s in *Wap* promoter driven GEMM vary widely from younger (21 weeks) to older (81 weeks) females. Examples include *Wap* promoter driving the expression of the intracellular domain of Notch4 with a longer T50 (43 weeks<) (nulliparous) (Gallahan et al., 1996), a truncated form of the SV40 T-antigen (*T121*) (Simin et al., 2004), SV40 large T (*Tag*) (Tzeng et al., 1993; Goetz et al., 2001), *TDGF1* (Sun et al., 2005), SV40 small T (*tAg*) (Tzeng et al., 1993; Goetz et al., 2001) and constitutively active rat *Mmp3* (Sympson et al., 1994).

Parity enhances tumorigenesis in the *Wap-Notch4* (Gallahan et al., 1996). *Wap-Shh* is a *Wap* promoter driven GEMM which does not develop parity-dependent tumors other than in *Cre* expressing GEMM (García-Zaragoza et al., 2012). Interestingly, *Wap-Myc* showed the shortest T50 (21 weeks) (Schoenenberger et al., 1988) amongst the *Wap*-promoter driven GEMM. In contrast, *MMTV-Myc* showed longer latency (T50 = 43 weeks) (Stewart et al., 1984; Cardiff et al., 1991). This difference could reflect the transient *Myc* expression in *Wap-Myc* versus the sustained expression of *Myc* in *MMTV-Myc*. These *Wap*- promoted GEMM with longer T50s may provide useful clues for comparison of parity-related human breast cancer (Albrektsen et al., 2005; Meier-Abt and Bentires-Alj, 2014) and the effects of lactation in reducing human breast cancer risk (Ursin et al., 2004). For example, *Wap-Myc* (or other *Wap*-promoter driven) mice can have induced pregnancy at different ages and analyzed for tumorigenesis to identify the mechanism of higher risk in the first pregnancy in older age. The experiment can also be expanded to identify the mechanisms of multiparity dependent-or breast feeding dependent-risk reduction for breast cancer.

## GKO mouse models and mammary tumors

GKO mice have also been used to analyze the molecular mechanisms in breast cancer. The *Trp53* (*p53*) deletion is best known GKO mouse that develops mammary tumors. It is based on the study of heterozygous deletions found in *Li-Fraumeni syndrome* (Donehower, 1996). *p53* heterozygous and homozygous knockout mice on mixed genetic backgrounds have tumor-free survivals (T50) of 78 and 18 weeks, respectively (Donehower, 1996). In contrast, on a pure Balb/c background *p53* heterozygote and homozygous knockout mice have tumor-free survivals (T50) at 54 and 15.4 weeks respectively (Kuperwasser et al., 2000). Although these *p53* GKO mice develop various types of tumor, palpable mammary tumors are rare on the mixed backgrounds. The Balb/c model had “mammary tumors” at the rate of 78%. However, microscopic examination of the masses revealed that 68% of “tumors” consisted of palpable masses were stromal abnormalities and sarcomas rather than epithelial tumors (Kuperwasser et al., 2000). Other studies with p53 deletions targeted to the mammary gland resulted in forming heterogeneous types of mammary tumors, suggesting a second event with different oncogenic drivers (Cardiff et al., 2006).

We reported that a signal transducer and activator of transcription 1 (*Stat1*) GKO in 129S6/SvEvTac background has a reduced branching morphogenesis during mammary gland development (Chen J. Q. et al., 2015). Most important, this GKO developed ER positive luminal-subtype mammary carcinomas with T50 at 91 weeks in nulliparous mice and 78 weeks in parous mice (Mori et al., 2017). This is a rare GKO which forms a morphologically uniform type of mammary tumor without crossing with the other GEMM or *p53* GKO. This unique GKO will be discussed in more detail below.

Some studies using GKO crossed with tumorigenic GEMM targeting mammary epithelium have been instructive. Cell-cell association is also a microenvironmental signal to epithelial cells and provides important signals to polarize epithelial cells. *Cdh1 f/f; Trp53 f/f* is a Cre-dependent conditional deletion of *E-cadherin* (*Cdh1*) and *p53* resulting in double GKO only in cells (and their progeny) that express the bacterial (exogenous) Cre recombinase. When crossed with *K14-Cre* (Derksen et al., 2006) or *Wap-Cre* (Derksen et al., 2011) to conditionally knockout *Cdh1* and *p53* in mammary basal epithelial cells or epithelial cells, respectively, the resulting *K14-Cre; Cdh1 f/f; Trp53 f/*+ developed mammary and skin tumors with T50 of 71 weeks. However, the homozygous deletions developed tumors with shorter T50s: *K14-Cre; Cdh1 f/f; Trp53 f/f* (T50 = 31 weeks) and *K14-Cre; Cdh1 f/*+ (or +/+)*; Trp53 f/f* (T50 = 48 weeks) (Derksen et al., 2006). In contrast, the *Wap* promoter versions of similar crosses, *Wap-Cre; Cdh1 f/f; Trp53 f/*+, formed mammary tumor with T50 of 83 weeks (calculated from Kaplan-Meier tumor free survival) which was much longer latency than *Wap-Cre; Cdh1 f/f; Trp53 f/f* (T50 = 28 weeks), *Wap-Cre; Cdh1 f/*+*; Trp53 f/f (T50* = *45 weeks), and Wap-Cre; Cdh1*^+/+^*; Trp53 f/f* (T50 = 42 weeks) (Derksen et al., 2011). In both *K14-Cre* and *Wap-Cre*, genotype with *Cdh1 f/f; Trp53 f/*+ exhibited prolonged latency, suggesting that these GEMM might be useful for the study of aging-related mammary tumorigenesis. *Cdh1* GKO model as another example of a homozygous GKO that is not sufficient for tumorigenesis but requires a cross with other GEMM to induce tumors.

Defects in *BRCA1 and BRCA2* genes have been recognized as risk factors for human breast cancer and ovarian cancer (Narod and Foulkes, 2004). *Brca1KO and Brca2KO* have been developed with deletions in different exons. Most of the homozygous *BrcaKO* (mutants of *Brca1KO* and *Brca2KO*) were embryonic lethal (Evers and Jonkers, 2006). Conditional KO models of exon11 of *Brca1* (*Brca1*^*Ko*/*Co*^: mouse carrying a *Brca1*-null allele *Brca1*^*Ko*22^ and a conditional allele *Brca1*^*Co*^ crossed with *WAP-Cre* or *MMTV-Cre*) (Xu et al., 1999) have been analyzed for BRCA1 related studies. Females of these *Brca1* GEMM do not form mammary tumors in the first 10 months of life. Within 10–13 months they do develop a tumor with low incidences of 2 of 13 *Brca1*^*Ko*/*Co*^*; WAP-Cre* and 3 of 10 *Brca1*^*Ko*/*Co*^*; MMTV-Cre*. These lower rates of tumorigenesis are increased and accelerated in crosses with *Trp53* heterozygote: *Brca1*^*Ko*/*Co*^*; MMTV-Cre; Trp53*^+/−^ mice (8 out of 11 between 6 and 8 months age) (Xu et al., 1999). The low incidence and prolonged latency suggest that these GEMMs could be useful for aging studies. However, like the majority of examples, to date these studies have not specifically emphasized “aging processes” in these models.

## Mouse strains and their basic characteristics

Comparison of mouse models should be done in same background strain because of the intrinsic variation of phenotypes in each strain. Backcrossing GEMM to adjust the strain requires time, effort, and money. Any combination of these factors can be the reasons for avoiding breeding to homozygosity. But why this is important? Hunter's classical study of the *MMTV- PyMT* in 20 different background strains document the effect of genetic background a single transgene raised in the same colony (Lifsted et al., 1998). The F1 crosses exhibited different tumor latencies and different metastatic potentials. Studies from other facilities show that *MMTV-PyMT* had a T50 in C57BL/6: *MMTV-PyMT* of 92 days (14 weeks) as compared to the shorter T50 (43 days; 7 weeks) in FVB: *MMTV-PyMT* (Davie et al., 2007).

The normal life span in each colony is critical for the evaluation of any age-related study. Each inbred mouse strain has a different life span. Studies from the Jackson Laboratory have reported the comparison of survival rates from 12 to 20 months. They showed that the survival rate in C57BL/6, 129S1/SvlmJ, and FVB/NJ are almost identical (Sundberg et al., 2011). However, the survival rate at 20 months is lower in FVB/NJ (Sundberg et al., 2011). Therefore, the longevity is variable in different mouse strains. Comparison of life span in 32 inbred strains showed life spans that vary with gender and strain. For example, FVB/NJ (female = 760 days and male = 591 days), 129S1/SvlmJ (female = 819 days and male = 882 days) and C57BL/6J (female = 866 days and male 901 days) are life span in these mouse strains (Yuan et al., 2009).

The cause of the death is also variable in inbred mice. Age-related diseases and causes of death typical of some common strains have been documented (Brayton et al., 2012). Not surprisingly, their comparison of end of life neoplasms in three strains (B6, 129 and B6,129) in three institutions (UW, NTCR, NIH) also reveal differences incidence of neoplastic and non-neoplastic disease. These differences could be attributed to difference in mouse housing and maintenance.

Genetic drift in isolation is another factor. For example, some but not all FVB colonies have pituitary abnormalities. Shorter life spans in FVB might be partially related to the pituitary gland abnormalities (pituitary tumor 32%, pituitary hyperplasia 14%) in aging FVB/N mice. The study showed that about 30% of aged (>80 weeks) females also developed mammary gland tumors (Radaelli et al., 2009). Since FVB have been the most commonly used strain for transgenic targeting of the mammary epithelium, investigators using this strain need to collect pituitary at the end of the experiment.

The Brayton's study lists the factors which can affect mouse phenotype and experimental outcomes. Difference in diet (calories, its contents), enrichment, housing (cage type, density/cage, and pathogen status), ear tag, infectious agents, light cycle, and temperature can influence all phenotypes and experimental outcomes (Brayton et al., 2012). For example, studies with *MMTV-neu* show that diet alters the percent tumor free animals (Yang et al., 2003; Liu et al., 2005) and efficacy of tamoxifen (Liu et al., 2005).

## The microenvironment

The normal mammary microenvironment of the mouse has been extensively studied. However, most reviews emphasize the immune system or hypoxia (Rothschild and Banerjee, 2015). With the success of immunotherapy syngeneic tumor transplant models have become extremely useful. Without a doubt, all the other cell populations deserve mention. Whereas many factors play major roles in the epithelial-environmental, there is almost no information with the lack of mouse models of microenvironmental factor and aging. As examples of some of the molecules, we discuss herein the metalloproteinases and adhesion molecules.

Extracellular proteins, cell surface molecules and signaling molecules are the targets for the analysis of stromal-epithelial interactions. Some molecules involved in association between extracellular matrices (ECMs) and cell surface receptors have been analyzed in GKOs. These GKOs are often crossed with tumorigenic *MMTV-LTR* or *Wap*-promoter driven GEMM, because GKO of these molecules might not be sufficient to form palpable mammary tumors by themselves. As summarized in Supplementary Data Sheet 1, ECM molecule-related GKOs have been crossed mostly with *MMTV-PyMT* and *MMTV-neu*.

*For example*, metalloproteinases (matrix metalloproteinase: MMP; a disintegrin and metalloprotease: ADAM) are a family of zinc binding type extracellular proteinases which process ECM molecules and surface molecules (Wolfsberg et al., 1995; Nagase and Woessner, 1999). These molecules are known to be involved in various physiopathological activities including development, tissue remodeling and cancer cell invasion (Rudolph-Owen et al., 1998; Seiki et al., 2003; Fata et al., 2007; Reiss and Saftig, 2009; Mori et al., 2013). Some of these MMP genes (*Mmp-8*, and *-14*) and ADAMs (*Adam-12* and -*ts1*) have been used in studies of mammary tumor progression by crossing GKO with *MMTV-PyMT*. Deletion of *Adam* genes delay *PyMT* tumor growth and metastasis (Fröhlich et al., 2011; Ricciardelli et al., 2011). Deletion of the *Mmp* genes had a different effect in *PyMT* tumorigenesis. Interestingly, both *Mmp8*KO and *Mmp14*KO in bigenic crosses with *PyMT* developed tumors earlier than WT-*PyMT* control (Szabova et al., 2008; Decock et al., 2015). Lung metastasis of *PyMT* tumor was reduced in bigenic mice with *Mmp14*KO (Szabova et al., 2008). In contrast, *Mmp8* deletion enhanced *PyMT* pulmonary metastasis (Decock et al., 2015).

Tissue inhibitor for metalloproteinase 3 (*Timp3*) GKO when crossed with *MMTV-PyMT* and *MMTV-neu* inhibited tumor growth and lung metastasis (Jackson et al., 2015). The T50 in *MMTV-neu* in the absence of *Timp3* was ~76 weeks compared with ~31 weeks in *Timp3*^+/+^; *MMTV-neu* (calculated from Kaplan-Meier curve of the age at first detection of tumor) (Jackson et al., 2015), suggesting that *Timp3*^−/−^ *MMTV-neu* might be another suitable model for studying aging-related breast cancer progression. Whereas *Timp3*^−/−^; *MMTV-PyMT tumor* delayed T50 (~13 weeks compared with control: 10 weeks), tumor growth was accelerated once tumor was formed (Jackson et al., 2015). Since TIMP3 has broad-spectral inhibition activity on both MMPs and ADAMs (Brew and Nagase, 2010; Murphy, 2011), TIMP3 target protease activities might be differentially involved in tumor growth and metastatic conversion. Once spatiotemporal distributions of MMPs, ADAMs, and TIMP3 were identified during mammary tumor progression are revealed, the TIMP3 effects on each target protease can be understood. Nonetheless, the delay in tumorigenesis in *Timp3*^−/−^; *MMTV-neu* could be another useful model for studying age-related mammary tumorigenesis.

Other GKO mice crossed with *MMTV-PyMT* and *-neu* have also been used to study the functions of cell adhesion molecules' involvement during the neoplastic process. In particular, many of these molecules mediate the association between cells and microenvironment. Since *PyMT* tumors forms earlier than *neu* tumors, the *PyMT* tumor model may not be as good a model for the study of aging. However, accelerating or delaying tumorigenesis in *neu*- or *PyMT*-tumor might reveal the function of target molecules that may affect aging.

The models described here are GKO crossed with other mammary tumor models. To analyze the association between collagen and the receptor (integrin a2b1), *Itga2*KO was developed (Chen et al., 2002). This *Itga2*KO was then crossed with *MMTV-neu* (Ramirez et al., 2011). The bigenic cross showed the earlier T50 (31 weeks) with higher metastatic levels then the comparable *Itga2*^+/+^; *MMTV-neu* control (33 weeks). CD151, a tetraspanin molecule associating with laminin binding integrins (Hemler, 2005), was knocked out and the mice crossed with *MMTV-neu* to study the molecular interactions with anErbB2 driven cancer (Deng et al., 2012). The T50 of the bigenic *CD151*^−/−^; *MMTV-neu* was 48 weeks which indicates slower tumor growth compared with the control 38 weeks in the control *CD151*^+/+^; *MMTV-neu* mice.

The GKO of *Hic5*, a focal adhesion scaffold/adaptor protein, was crossed with *MMTV-PyMT* and the T50 was 9 weeks longer than the heterozygous knockouts with *Hic5*^+/−^; *MMTV-PyMT* (Goreczny et al., 2017). Protein-tyrosine phosphatase 1B (PTP1B), which is involved in regulating signals between cell and microenvironment, was knocked out and crossed with *MMTV-PyMT* and *-neu* (Bentires-Alj and Neel, 2007). Interestingly, *Ptp1b*^−/−^*; MMTV-neu* exhibited a greatly reduced tumorigenesis of *neu* tumor (27% mice formed tumors at 69 weeks) as comparing with *Ptp1b*^+/−^*; MMTV-neu and Ptp1b*^+/+^*; MMTV-neu* (T50 = 61 weeks and 57 weeks respectively). *Ptp1b*^−/−^*; MMTV-PyMT* did not, however, significantly delay PyMT tumor growth (Bentires-Alj and Neel, 2007). Some mammary tumor forming GEMM are also used to inhibit target molecule's activity (MMP9, Lysyl oxidase) or crossed with transgenic of mutant protein (e.g., collagenase resistant type I collagen alpha1 chain, Col1a1) are indicated in Supplementary Data Sheet 1.

These mouse models are useful for the study of various aspects of tumor progression. The mammary promoters (*MMTV-LTR, Wap*-, and *K14*-), however, are activated in other cells in different organs. *MMTV-Cre; Gt(ROSA)26Sor* transgenics demonstrate that the *MMTV-LTR* promoter actively transduces Cre in a variety of non-mammary tissues (e.g., salivary gland, skin, seminal vesicles, lymphocytes, megakaryocytes, erythroid cells, etc.) (Wagner et al., 2001). In addition, three *MMTV-Cre* promoted lines showed the defects in nursing pups; line A showed impairment in lactation, line F completely failed to nurse, and line D was normal.

*Wap-cre; Tg(CMV-nlacZ)1Pgr* showed Cre expression in brain, muscle and testes, and one line had Cre expression in mammary gland and the brain (Wagner et al., 1997). *K14*-*Cre* (Vasioukhin et al., 1999), *K5-Cre* (Tarutani et al., 1997), and *Lgr-CreERT2* (Kinzel et al., 2014) have also been used to conditionally knockout gene targets in mammary epithelial cells (Taddei et al., 2008; Koren et al., 2015; Liu et al., 2016). These promoters were originally developed to study skin tissue (Tarutani et al., 1997; Vasioukhin et al., 1999; Kinzel et al., 2014) and other organs (gut and kidney) (Kinzel et al., 2014). Thus, these promoter activities are not specific to mammary epithelial cells. This suggests that the effect on mammary gland in GKOs crossed with these Cre expressing GEMM might be more complex than mammary epithelial-specific promoters. Once aging dependent or independent inflammatory activity occurs in one organ, the activity of immune cells will be modified and can affect other organs in the same animal. In addition, crossing these models with different mouse background strains can cause the variations in phenotypes (e.g., immune cell activity, drug efficacy, tumorigenesis). In next section, we summarize these parameters.

## Aging in immune cells

The Jackson Laboratory's analysis of immune cell populations in peripheral blood in 6 months old inbred mice demonstrates that lymphocytes in female (F) and male (M) differ between strains and genders (Petkova et al., 2008). Examples include C57BL/6J which are F: 71.5% and M:78.2%, FVB/NJ are F:79.9% and M:52.5% and 129S1/SvlmJ are F:49.4% and M:60.5%(Petkova et al., 2008) Monocytes in C57BL/6J are F:4.47% and M:7.62%, FVB/NJ are F:5.79% and M:6.6% and 129S1/SvlmJ are F:8.3% and M:8.61% (Petkova et al., 2008). More importantly, these percentages of immune cells and the immune cell activities might also be different with age of the mouse strain. Studies of aging in immune cells, named “*inflammaging*” (Baylis et al., 2013; Salvioli et al., 2013; Franceschi and Campisi, 2014; Franceschi et al., 2017), indicate that each immune cell lineage and their activities change with age. These age-related changes can directly affect tumor initiation and progression. Since immune cells are a factor in “tumor microenvironment” and important in clearing damaged cells (such as senescence cells), immune cell activities in aged mice might also provide hints to decipher the link between aging and breast cancer.

## Lymphocytes

Decreased lymphoid progenitors and involution of the thymus occur during aging (Linton and Dorshkind, 2004; Palmer, 2013). This results in a decrease in T-cell output and shifts in cell types from naïve (CD62Lhigh CD44low) to memory T-cells (CD62Llow CD44high) (Shimatani et al., 2009; Sprent and Surh, 2011; Goreczny et al., 2017). These aging memory T-cells are PD1+ and CD153+CD44high CD4+ (Sato et al., 2017). They have the same phenotype as senescence associated T-cells found in lupus pathogenesis (Tahir et al., 2015), suggesting that the aging dependent increase in CD4+ T-cells might also be involved in increased risk of autoimmunity. Memory CD8+T cells are also increased in aged mice (Chiu et al., 2013). The memory CD8+ T cells in older mouse expand the numbers of effector CD8+ T cells in LCMV infection compared with younger mouse derived memory CD8+ T cells (Eberlein et al., 2016). This critical change in T cells could be an anti-pathogen defense system in aged mice with less naïve T-cells.

In aged humans, decreased memory B-cells and increased naïve B-cells are observed (Chong et al., 2005). In mice, B-cells numbers are decreased with aging because of decreased precursors in bone marrow (Riley et al., 1991). This is further defined as the defect in the process between pro-B and pre-B maturation in aged mice (Stephan et al., 1996). The defect in pro-B to pre-B maturation in aged mice is attributed to insufficient levels of IL-7 (Fleming and Paige, 2001). In *MMTV-PyMT* mice, splenic B-cells are increased, This might be considered as evidence of inhibitory factors secreted from the PyMT tumor cells that inhibit B-cell progenitor proliferation and maturation (Moreau et al., 2016).

Regulatory T cells (Treg: a.k.a. suppressor T cells) are also altered in aged mice. CD4+/CD25+/Foxp3+ and CD8+/CD25+/Foxp3+ Treg cells are almost two times higher in spleens and lymph nodes from aged mice, and prevents the activation of immune response (Sharma et al., 2006). Increased Treg are also observed in both peripheral blood and tumor microenvironment of invasive breast cancers (Liyanage et al., 2002). C57BL/6 with *Foxp3* knocked-out/knock-in human diphtheria toxin receptor (DTR; *Foxp3*^DTR^) (Kim et al., 2007) is a GEMM for analyzing Treg activities in the presence or the absence of diphtheria toxin (DT). Orthotopic injection of a C57BL/6; *MMTV-PyMT* derived mammary tumor into *Foxp3*^DTR^ treated with DT results in reduction of PyMT tumor growth. Enhanced apoptosis was noted with proliferation of naïve CD4+ cells and CD8+ cells (Bos et al., 2013).

## Macrophages

Macrophage phagocytosis is involved in clearing apoptotic cells and is important for maintaining tissue homeostasis. However, aging reduces the macrophage capacity for clearing apoptotic cells (Aprahamian et al., 2008). Moreover, macrophage treated with tunicamycin, an inducer for endoplasmic reticulum stress, showed that macrophages isolated aged mice have more apoptosis compared with macrophages from younger mice (Song et al., 2013). Macrophage activation is also altered in aging. Cytokine induction to direct macrophage to M1 is increased in aged mice, but the M2 induction is decreased. Conversely, induction to M1 is weaker in younger mice, but induction to M2 is stronger (Lee et al., 2013).

Deletion of p53 in hepatic stellate cells (HSCs) induces fibrotic liver tumors which have proliferating HSCs and senescence of HSCs (Lujambio et al., 2013). Interestingly, conditioned media from proliferating HSCs induces M2 polarization, and contrary, conditioned media from senescent HSCs induces M1 polarization of macrophage (Lujambio et al., 2013). The macrophage polarization in two studies suggest that aging reduces macrophage induction to M2 status. This might decrease the anti-tumor activity of macrophages and facilitate tumorigenesis. This secretion from senescence cells is called senescence associated secretory phenotype (SASP). SASP causes pro- and anti-tumor activities through affecting to different types of immune cells (Rao and Jackson, 2016). Since secretion from cells have soluble factors (e.g., cytokines, processed proteins from cell surface) and extracellular vesicles (exosomes, microvesicles, and apoptotic bodies) (El Andaloussi et al., 2013), the identification of the contents within each fraction, function, levels, and distribution is now required to understand SASP as a part of aged microenvironment.

## Myeloid derived suppressor cells (MDSCs)

MDSC is a key immune cell in the control of immune cell activities and reciprocally associates with Treg to regulate tumor progression (Hurez et al., 2012, 2017; Marvel and Gabrilovich, 2015). MDSCs are increased in aged mice (Grizzle et al., 2007; Enioutina et al., 2011) and human (Verschoor et al., 2013). This age dependent MDSC expansion is regulated by Nf-kB activity in aged mice (Flores et al., 2017). Aged mice have increased MDSCs and less T-cell cytotoxicity compared with young mice. The suppression of T-cell cytotoxicity is due to the high levels of arginase activity in aged MDSCs (Grizzle et al., 2007). Interestingly, the increase of MDSCs is also observed in bone marrow and spleen of tumor-bearing mice compared with naïve mice (Huang et al., 2006). These MDSCs from tumor-bearing mice induce the development of Treg (Huang et al., 2006) and T-cell tolerance (Kusmartsev et al., 2005; Huang et al., 2006). These age-related tumor permissive activities of MDSCs have been targeted for immunotherapy. Higher levels of tumor permissive activity in aged mice can be reduced with anti-GR1 targeting MDSCs, which reduces melanoma tumorigenesis (Hurez et al., 2012). Aged MDSCs have higher levels of B7-H1 (a.k.a. Programmed death ligand 1, PD-L1) induced by IL-10 activity, and the inhibition of B7-H1 reduced the tumorigenesis of Lewis lung cancer cells in aged mice (Chen S. et al., 2015).

## Dendritic cells

Dendritic cells (DCs) are antigen-presenting cells and categorized by differentiation status into conventional DCs (cDCs), plasmacytoid DCs (pDCs), and monocyte DCs (moDCs). The DCs are involved in a key function on T-cell activation (Gardner and Ruffell, 2016). Twenty six-month-old male C57BL/6 mice (from Jackson Laboratory and maintained at Oregon State Univ) had decreased splenic pDCs and CD8+ cDC (Wong et al., 2010). Another study with 18-month-old female C57BL/6 mice (from State Foundation for Production and Health Research and maintained at Instituto de Pesquisas Biomédicas, Brazil) found reduced numbers of CD11c+ cells but only cDCs were significantly reduced as compared with young mice (Pereira et al., 2011). However, C57BL/6 mice (from Animal Resources Center and maintained at The Centenary Institute for Cancer Medicine and Cell Biology, Australia) older than 18 months had increased cDCs in spleen and lung as compared to young mice. In contrast, Balb/c mice did not show the same difference between aged and young mice (Tan et al., 2012). This study also showed by comparison of T-cell activation of DCs from aged- and young- C57BL/6 mice no differences in co-stimulatory molecules (CD80, CD40, and CD70) except for CD86 (B7-2) which is reduced in aged cDCs with influenza infection (Tan et al., 2012). However, aging effect on CD86 levels are not differ in cDCs in above mentioned two other studies (Wong et al., 2010; Pereira et al., 2011) and without influenza infection (Tan et al., 2012). Thus, the difference in cDCs might be affected by variations in mouse strain background, gender, age, housing environment and pathogen activities. In a ovarian tumor model (C57BL6: *LSL-K-rasG12D/*+*p53*^*loxP*/*loxP*^ generated *from Kras*^*tm4Tyj*^ and *Trp53*^*tm1Brn*^ *mice*), DCs changed the phenotype from immunosuppressive in the earlier stage of tumor expansion to immunogenic in the advanced stage of cancer (Scarlett et al., 2012), This switch suggests that the status of DCs might indicate the checkpoint for neoplasia as it become malignant.

## Natural killer cells

Natural killer (NK) cell is a type of lymphocyte and an important cell for innate immunity.

A comparison of NK cells in healthy people from 4 to 106 years of age showed that NK cells were increased in older age, but NK activity in middle-aged subjects was weaker compared to young subjects and to centenarians (Sansoni et al., 1993). In mice, there were strain dependent and gender variations in a percentage of peripheral NK cells (Petkova et al., 2008). Whereas C57BL/6 and 129S1/SvlmJ mice have similar percentages of NK cells between the genders (C57 female: 3.74%, male: 4.02%; 129 female: 5.5%, male: 4.66%), FBV/NJ male mice have a significantly fewer NK cells (3.49%) than females (7.15%) (Petkova et al., 2008). NK cells are increased in bone marrow in aged Balb/c mice, resulting in decreased pro-B cells in aged animals (King et al., 2009). NK cells (with different levels of CD56 and CD16) were increased in the peripheral blood from breast cancer patients with advanced cancer but were mostly immature and non-cytotoxic subsets (Mamessier et al., 2013). Spleen tissues in FVB; *MMTV-neu* mice have almost similar number of NK compared with wild type (WT) FVB mice while other immune cells (T cells, DCs, MDSCs, and Tregs) are increased in FVB; *MMTV-neu* (Abe et al., 2010). In C57BL/6; *MMTV-PyMT*, tumor derived NK cells are immature compared with splenic NK cells. An adaptive transplantation of splenic NK cells into tumor resulted in decreased NK maturation, suggesting that tumor microenvironment in *MMTV-PyMT* tumor determines the NK cell maturation. The authors suggested that IL12 and TGFb are involved in NK maturation (Krneta et al., 2016). Thus, although NK cell numbers and activities differ in human and mouse, levels of immaturity in NK cells is similar in human breast cancers and mouse mammary tumors.

Immune cells' variations and activities different between mouse strains, genders, and age. The spatial density and activity of each type of immune cell within the tissue is critical to understanding associations between immune cells and immune targeted cells (e.g., tumor cells and cells in senescence). These associations are important for understanding age related tumorigenesis. They are equally critical for understanding the process of immunoediting from “elimination,” “equilibrium” to “escape” (Schreiber et al., 2011; Mittal et al., 2014). The challenge is now to relate the two phenomena.

## Aging in the human breast and mouse mammary gland and microenvironment

Human breasts and mouse mammary glands are functionally similar. However, these tissues have distinct structural differences especially during the aging process (Cardiff et al., 2018). In puberty, human breast completes the formation of branching collecting ducts with terminal duct lobular unit (TDLU) in collagen fiber rich stroma. In contrast, mouse mammary glands form a ductal branching tree without TDLU structures and primarily embedded in adipose tissue. Mouse mammary gland develops lobulo-alveolar structures with pregnancy. The murine lobulo-alveolar units quickly involute within a week of weaning (Cardiff and Wellings, 1999). Human breast also expands lobulo-alveolar units with endocrine signals during pregnancy and parturition. In contrast to the mouse, the human involution is slow and the TDLU structures remain (Cardiff and Wellings, 1999) with some structural variations, which correlate with time, after the postpartum (Jindal et al., 2014). Age- related involution in human breast occurs during perimenopause (or surgical- or chemical induced menopause) with the atrophy of senescence, which results in major reduction in TDLU structures (Cardiff and Wellings, 1999; Cardiff et al., 2018). In contrast, aged mice continue to ovulate and have estrus cycles. This limits the use of mice to model human peri- and post-menopause conditions (Nelson et al., 1982; Finch et al., 1984). In order to analyze menopause dependent effects, the investigator can us surgery- and chemical treatment-dependent induced menopause in mice. One approach might be ovariectomy (Finch et al., 1984) which quickly shuts down the 17β-Estradiol levels, stops the cyclicity and quickly maximizes FSH levels (www.jax.org). Another is 4-vynylcyclohexene diepoxide (VCD) treatment, which slowly increases FSH levels and slows down cyclicity (Brooks et al., 2016).

## Studying parity-related breast cancer and aging

Although multiple-pregnancies reduce the incidence rate of human breast cancer (Albrektsen et al., 2005), the risk reduction is sustained longer in women having a first pregnancy after 35 years old (Albrektsen et al., 2005; Meier-Abt and Bentires-Alj, 2014). Women having a first pregnancy after 30 years old also have higher risk for breast cancer and the risk is sustained higher until 65 years old compared with woman with nulliparous status (Albrektsen et al., 2005; Schedin, 2006; Meier-Abt and Bentires-Alj, 2014). The many factors (stem cells, hormone and hormone receptor status, growth factors, cytokines, ECM, and immune cell activities) which might be involved in these age- and parity-dependent breast cancer are discussed elsewhere (Schedin, 2006; Meier-Abt and Bentires-Alj, 2014).

The duration of breast-feeding is also an important factor. Prolonged nursing (lactation) reduces the risk of breast cancer even in aged cohort (50–64 years old)(Ursin et al., 2004). Analyzing involvement of each factor which functions as cancer promoting or suppressing will be a challenge for future research. The effects of parity have been well-documented in mouse models. Although some GEMM have prolonged tumor latency, factors relating to aging have not been recorded in many of the candidate models. But the current GEMM or newly generated GEMM combined with analysis indicated in this manuscript might give a hint for modeling the experiments. For example, the analysis of microenvironmental factors in parity facilitated, syngeneic orthotopic transplants in mice (Maglione et al., 2004; Namba et al., 2004, 2005; Borowsky et al., 2005) under different well-controlled conditions of age, parity status, parity cycles, and milk feeding duration. Since tumors from some GEMM grow too aggressively, tumors with slower tumor kinetics will be more suitable for analyzing aging and parity related tumorigenesis.

## *Stat1* GKO as a model for analysis for age related mammary tumorigenesis

The focus on mammary cancer in mice has been, quite correctly, the mechanisms that make the neoplastic epithelial cell autonomous. The transgenic mouse has provided the “Koch's Postulate” for modern genomic science and cancer by defining the neoplastic potential of numerous genes and/or characterizing genes that modify neoplastic progression. In spite of a plethora of these mice, they have not been used to address the issue of aging in breast cancer. Although research on the cancer microenvironment has blossomed, the issue of aging in GEMM has not been effectively addressed.

We have studied in detail a GEMM that includes prolonged tumor latency, 129S6/SvEvTac: *Stat1*^*tm1Rds*^ (129: *Stat1*^−/−^) mice (Meraz et al., 1996), which is a unique GKO that rarely develops mammary cancer before 1 year and has a T50 of 91 weeks (Mori et al., 2017). The *Stat1*^−/−^ females were held as long as 120 weeks (840 days) which exceeded the expected 817-day life span of wild type 129 females. From this perspective, the T50 was reached at 78% of the total life span. The 129: *Stat1*^−/−^ provides some insight into many of the factors considered in the review above and the opportunity to consider the complex issues in such models.

Other groups have demonstrated that deletion of *Stat1* leads to an increased susceptibility to mammary tumors (Klover et al., 2010; Raven et al., 2011; Schneckenleithner et al., 2011; Chan et al., 2012). The 129: *Stat1*^−/−^ model forms mammary tumors with a T50 at 91 weeks in nulliparous and at 78 weeks in parous females, similar to the report from the colony in Washington University (Chan et al., 2012). Mice younger than 32 weeks old in our colony did not have detectable neoplastic histopathology. Mice older than 52 weeks nulliparous without tumors had microscopic MIN (mammary intraepithelial neoplasia; 3/32 cases), and 23/32 cases had preneoplastic MIN or tumors.

Tumor-bearing nulliparous (9/13 cases) showed lobulo-alveolar hyperplasia even without pregnancy or exposure to the pregnancy-associated hormones and these mice had dilated mammary duct filled with milky white fluid. The older tumor-bearing nulliparous, with stunted growth in mammary gland (4/32 cases) still had underdeveloped ductal network (Chen J. Q. et al., 2015).

The strain variation is also an important issue in *Stat1* GKO. Other *Stat1* GKO lines, *Stat1*^*tm1Dlv*^, backcrossed into Balb/c strain also shows tumorigenesis in multiparous *Stat1-null* condition with mammary tumor and disease onset at 57 weeks which is shorter latency compared with *Stat1*^+/+^ (69 weeks) (Durbin et al., 1996). Balb/c: *Stat1*^*tm1Dlv*^ crossed with Balb/c: *MMTV-Neu* shows shorter latency of *Neu* type tumors as compared with Balb/c: *MMTV-Neu* nulliparous mice (42 vs. 50 weeks) (Raven et al., 2011). Similarly, *Stat1f/f* was crossed with FVB: Tg(MMTV-Erbb2^*^,-cre)1Mul (Ursini-Siegel et al., 2008) to conditionally knock out *Stat1* gene and simultaneously transduce *neu* in mammary epithelial cells, which also results in an earlier T50 in the *Stat1-null* condition (50 vs. 63 weeks) (Klover et al., 2010). These two studies (Klover et al., 2010; Raven et al., 2011) suggest STAT1 as a tumor suppressing molecule, and the work of Klover et al. (2010) indicates that STAT1 function in mammary epithelium is anti-tumorigenic.

Interestingly, mammary gland development in *Stat1* GKO is only impaired in 129S6/SvEvTac (Chen J. Q. et al., 2015) but the defect has not been observed in GKO in the Balb/c background (Schneckenleithner et al., 2011). Mammary tumorigenesis is also different in these two strains: 129:*Stat1*^−/−^ forms homogeneous mammary tumors with or without parity (Chan et al., 2012; Mori et al., 2017), but Balb/c: *Stat1*^−/−^ formed heterogeneous tumors only with multiparity (Schneckenleithner et al., 2011). However, these animals may not have been maintained long enough to develop tumors. For example, in one study, the Balb/c: *Stat1*^−/−^ females developed mammary tumors only after being force-bred seven times.

The interpretation of these observations is compounded by the use of different molecular constructs for the knockout of *Stat1*, which further could have influenced the outcome. The Meraz construct deletes the N-terminus of STAT1 in 129/Sv mice, whereas residual low expression level C-terminus fragment remains (Meraz et al., 1996). The other construct deleting exon 18–20 in unknown background mouse strain does not have this fragment detected by anti-C-terminus STAT1 (Bailey et al., 2012). However, exon 18-20 deleted *Stat1*^−/−^ mice still need to be investigated with anti-N-terminus STAT1 and analyzed for any defect. Thus, mechanisms leading to tumor development in 129: *Stat1*^−/−^ remain unclear.

The initial study of normal mammary development in the 129: *Stat1*^−/−^ females revealed a defect in branching morphogenesis accompanied by abnormal terminal end buds (Chen J. Q. et al., 2015). Given the type of molecular signaling, this could be expected. The DeOme's “Test-By-Transplantation” (Deome et al., 1959) with reciprocal transplants into gland-cleared mammary fat pads was used. The GKO epithelium developed normally in wild type mammary fat pad. Thus, the developmental phenotype was not intrinsic to the mammary epithelium.

Subsequent experiments proved that the GKO fat pad lacked cytokines needed for growth. Therefore, we concluded that the microenvironment of the fat pad was responsible for the growth defect. Interestingly, the GKO fat pad could be stimulated to produce adequate levels of cytokines using injections of a progesterone and prolactin. Unfortunately, we were unable to perform similar experiments using fat pads from older GKO females. These experiments, however, point to the importance of the microenvironment and the endocrine system in mammary development and demonstrate the consequences when they are defective.

The 129: *Stat1*^−/−^ mammary tumors are morphologically and molecularly unique, ovarian dependent tumors. The tumors involve the neoplastic transformation of a stem cell population that is FoxA1+, ER+, PR+ with unique atypical cells with large, oval, moderately pleomorphic nuclei with an open chromatin and prominent nucleoli with pale cytoplasm, named large oval pale (LOP) cells. The characteristic of LOP cell is similar to “committed progenitor cells” in mouse mammary gland (Smith and Medina, 1988; Chepko and Smith, 1999) and suprabasal clear human breast cells (Toker, 1967; Stirling and Chandler, 1976, 1977; Smith et al., 1984). In addition, FoxA1 is known as a key factor (“pioneer transcription factor”) (Zaret and Carroll, 2011; Jozwik and Carroll, 2012; Zhang et al., 2016) which regulates the expression of estrogen receptor alpha (Bernardo and Keri, 2012; Liu et al., 2016; Zhang et al., 2016) and is an essential factor for mammary branching morphogenesis (Liu et al., 2016). The cell of origin is readily identified as the LOP cell. In the early manifestations, the LOP cell can be found distributed along ducts. Later, the LOP cell forms large, invasive neoplasms. The epithelial intrinsic mechanisms have been described including activation of the JAK2 axis, deregulation of SOCS3 and mutation of the prolactin receptor (Chan et al., 2014; Griffith et al., 2016).

The differences between two GKO *Stat1*-null strains might be reflecting the differences in immune cells: percentages of CD4+ T cells, CD8+ T, cells and NK cells in peripheral blood are higher in Balb/cByJ, and granulocytes, eosinophils, monocytes and B-cells are higher in 129S1/SvlmJ (Brayton et al., 2012). Because of STAT1 involvement in interleukin 2 signal in T lymphocytes and NK cell lines (Frank et al., 1995), the *Stat1*^−/−^ mice were originally generated to analyze the STAT1 involvement in NK cell activity via interferon (IFN) dependent signals and in other cytokines' signals (Meraz et al., 1996). The study showed that STAT1 deficiency leads to inactivation in IFNa and IFNg signals (Durbin et al., 1996; Meraz et al., 1996) and has a defect in responding to microbial pathogens (Meraz et al., 1996) and viruses (Durbin et al., 1996; Meraz et al., 1996), but the response for other signals (growth hormone, EGF and IL-10) were not impaired (Meraz et al., 1996). In later study, 129: *Stat1*^−/−^ was shown to have a defect in cytolysis activity in NK cells (Lee et al., 2000).

Our pathological analyses of the three cohorts of older 129: wild type (WT), tumor free 129: *Stat1*^−/−^ and tumor-bearing 129: *Stat1*^−/−^ revealed characteristic lesions of aging: including. ovaries with luteinized stroma and fewer Graafian follicles. However, the hormone regulating glands (adrenal gland, thyroid gland, and pituitary gland) were all histologically normal. Evidence active estrus cycle was still found in 96 weeks old mice, indicating the ovarian hormones were still active. Although some 129: *Stat1*^−/−^ showed the defects such as ovarian cysts, not all female with ovarian cysts developed mammary tumor, and one tumor-bearing female had histologically normal ovaries. These observations on ovaries and estrus cycle indicate that the tumorigenesis in 129: *Stat1*^−/−^ is not due to the lack of ovarian hormones.

To understand the effect of the microenvironmental factors in *Stat1*-null tumorigenesis, syngeneic orthotopic transplants of *Stat1*-null tumor and MIN tissue into younger 129 WT and KO were performed. Tumors were palpable earlier in WT host. However, the tumor growth rates were almost the same in both hosts. This still resulted in 10-100 folds larger tumor in WT at the termination time point.

The analysis primary tumors in 129:*Stat1*^−/−^ showed that stroma adjacent to the tumor had larger infiltrates of inflammatory cells (macrophage and T-lymphocytes) with fibrosis as compared to other GEMM (Cardiff et al., 2006). These observations of immune cell activities suggested that *Stat1*-null tumor makes an immune-attractive tumor-adjacent microenvironment and has an “excluded infiltrate” phenotype (Hegde et al., 2016). To investigate the possible mechanisms, we analyzed factors secreted from *Stat1*-null cell lines and found that soluble factors inhibit macrophage migration whereas the extracellular vesicle fraction stimulates macrophage migration. This indicates that SASP activity in *Stat1*-null tumors involves with cross talk with the microenvironment.

The point of introducing our study here is to provide an example of analyzing aging- related tumorigenesis in the mammary gland. First, as we discussed in above, environmental factors (housing, cycles of circadian rhythm controlled by light/dark cycles, diet, viral- and bacterial-infections and any other pathogens) for maintaining mouse colony can affect mouse phenotype. It is not easy to adjust these parameters in every institution to compare mouse models, at least checking viral- and bacterial-infection should be done to consider if immune cell activity is different or not in each mouse colony. Our colony ismonitored using a serology profile (UC Davis mouse level2 serogenic profile: Mouse Hepatitis Virus, Sendai, Pneumonia Virus of Mice Reo-3, MPV, Minute Virus of Mice (parvovirus type) NS-1, M.pul and arth, Theiler's Murine Encephalomyelitis Virus part of GDVII strain, Reo-3, Lymphocytic Choriomeningitis Virus, Epidemic Diarrhea of Infant Mice Virus, Mouse Adeno DNA Virus 1 and 2, Mouse Noro Virus). In addition, bacterial pathogens are tested on cecum or nasopharynx and pinworms or fur mites are also routinely checked (Mori et al., 2015). In our case, although specific immune cell activities are not compared, 129: *Stat1*^−/−^ at UC Davis and Washington University showed identical mammary tumorigenesis phenotypes, especially for T50 in nulliparous and parous dependent and tumor subtypes (Chan et al., 2012; Mori et al., 2017).

Secondly, wild type and KO were necropsied to find causative factors of mammary tumorigenesis and cause of death (COD) especially for evidence associated with aging. The guidelines of for determination for of COD contributing to COD (CCOD) or cause of morbidity are as suggested by The National Center for Toxicological Research (NCTR), which is the important for aging studies (Kodell et al., 1995; Brayton et al., 2012).

Third, syngeneic orthotropic transplantation was used to understand some microenvironmental effects. Since many of GEMM have targeted gene regulation for embryonic stages or hormone dependency or inducible gene expression system multiple cell types could be targeted in these GEMM. In our case, *Stat1* gene deletion could affect any *Stat1* expressing cells. Therefore, the tissue microenvironment developed abnormally, or the tumor cells generated a unique microenvironment, or gene modification affected the microenvironment in *Stat1* GKO. Some the non-epithelial effects can be eliminated using syngeneic orthotopic transplantation. This approach worked very well for the early experiments with younger host. Unfortunately, only young hosts were available for transplantation. Testing hypothesis in aged host mice will have to held for our future experiments. Syngeneic orthotopic transplantation is also useful for studying spatiotemporal factors in aging or cancer research.

Fourth, the understanding of targeted aging or microenvironmental factors in mammary tumorigenesis, can be analyzed applying these techniques. These can provide a broad-context picture of the biology of aging and cancer.

## Summary

GEMM have been generated for analyzing functions of a gene in physiopathological events and are often crossed with other GEMM to further investigate context dependent biological phenomena. In breast cancer research, GEMM made for analyzing the gene involvement for mammary tumorigenesis have been used to study tumor progression in mammary glands and metastasis. Although a longer tumor latency (T50 value) suggests involvement of the target molecule in aging-dependent mammary tumorigenesis, few studies have defined how aging and impacts tumor initiation or tumor progression. This lack research effort might be explained by multiple issues: difficulties in separating the aging effect from tumorigenesis; the commitment of time to complete a project; and the cost and the effort required to model aging are all confounding. Tumorigenesis in most GEMM is too rapid to permit aging related comparisons. Complex parameters must be considered to understand entire *in vivo* biological system. Finally, lack of good GEMM for studying aging and mammary tumorigenesis simultaneously has hindered progress in this area.

129: *Stat1*^−/−^ is a model for the study of age-related mammary tumorigenesis. The frequency of tumorigenesis, T50 and tumor subtype of the model provides a model that is an age-related ER+ tumor model. The origin and the fate of LOP cells needs to be identified and coupled with immunoediting and microenvironment factors. One envisions that experiments with this model will provide a complex but informative picture of breast cancer. This will be major challenge for investigators including us but will lead to much sounder understanding. Fortunately, these issues are not limited to the 129: *Stat1*^−/−^ model but also applicable to many other GEMM.

The challenge is to understand the complex biology in each GEMM. It very important to share experimental information, data, and materials in addition to each publication. This will facilitate collaborative research by reducing time, cost and mouse usage and lead to an improved understanding of the biology of cancer in aging.

## Sharing GEMM derived information and materials on aging

Databases available for sharing information:

Genome informatics (MGI: http://www.informatics.jax.org/), Mouse tumor biology database (http://tumor.informatics.jax.org/mtbwi/index.do), European mutant mouse pathology database (http://pathbase.net/), National Cancer Informatics Program (NCIP) hub for a collaboratory for cancer research (https://nciphub.org/). These databases give basic information of registered mouse models. However, each institution's environmental information for maintaining mouse colony and serogenic profile for each GEMM is not well-documented. Available tissue samples with detailed conditions have not been organized. There is an activity of “*Sharing Experimental Animal Recourses, Coordinating Holdings*,” which suggests sharing the materials from experimental animal models to reduce animal use and accelerate collaborative research. Especially for sharing aging research models, sharing hub website has been developed (www.sharmuk.org). Our website, Center for Genomic Pathology, additionally provides online courses for educating researchers (www.ctrgenpath.org). These are important to further understand each GEMM characteristics by comparing with a certain condition, which facilitate the cancer and aging related research.

## Author contributions

HM and RC: Conceptualized this review article, preparing the manuscript; AB: Preparing the manuscript.

### Conflict of interest statement

The authors declare that the research was conducted in the absence of any commercial or financial relationships that could be construed as a potential conflict of interest.

## Supplementary material

Supplementary Data Sheet 1 Excel files are summarizing GEMM with following categories. Page1: a list of *MMTV-LTR* driven mouse models, page2: a list of *Wap*-promoter driven mouse models, and page3: a list of GKO and other models. Each mouse model is indicated as Symbol (registered name for mouse model), name (transgene information and registered person's name), background strain, mammary tumor, latency, and estimated T50. A list of GKO has PMID or PMCID as additional information.
